# Supplementary material for: Transcription Factor Lmx1b Negatively Regulates Osteoblast Differentiation and Bone Formation
Source: Int J Mol Sci. 2022 May 7;23(9):5225. doi: 10.3390/ijms23095225 (PMC9103437; doi:10.3390/ijms23095225)
Supplement: Supplementary file 1 [file ijms-23-05225-s001.zip › ijms-1701402-supplementary.pdf]

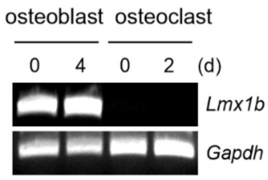

Figure S1. Expression of *Lmx1b* in osteoblasts and osteoclasts. Osteoblasts or osteoclasts were cultured in osteogenic medium or osteoclastogenic medium, respectively, for the indicated times. Expression of *Lmx1b* and *Gapdh* was determined using RT-PCR.

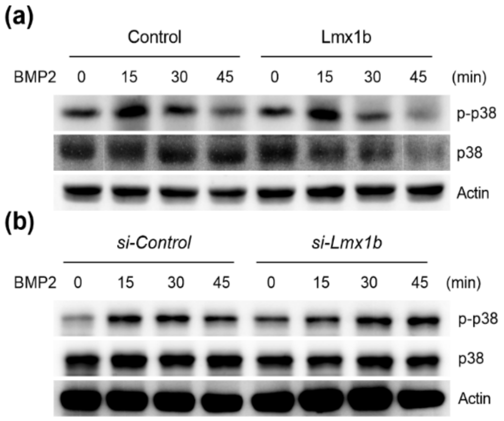

Figure S2. Lmx1b regulates the BMP2 signaling pathways in osteoblasts, with the exception of p38 MAPK activation. Lmx1b-overexpressing primary osteoblast precursor cells (a) or Lmx1b-silenced primary osteoblast precursor cells (b) were stimulated with BMP2 for the indicated time durations. Whole-cell lysates were subjected to western blot analysis with specific antibodies, as indicated.

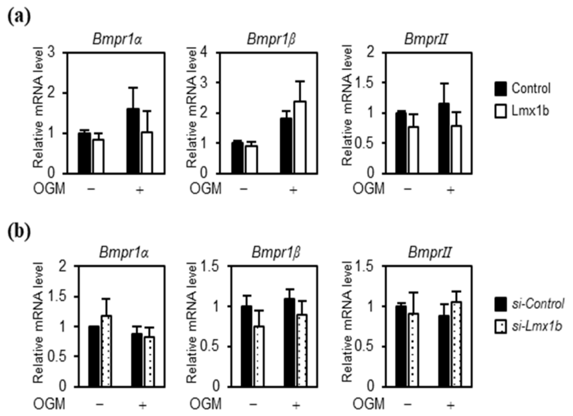

Figure S3. Lmx1b did not alter the expression levels of BMP receptors (BMPRs). Lmx1b-overexpressing primary osteoblast precursor cells (a) or Lmx1b-silenced primary osteoblast precursor cells (b) were cultured in osteogenic medium for 3 days. Total RNA was subjected to real-time PCR analysis of the expression of *Bmpr1α*, *Bmpr1β*, and *BmprII*.
